# Considerations for a Social Media Physical Activity Program: Exploratory Study

**DOI:** 10.2196/26008

**Published:** 2022-02-14

**Authors:** Katherine Q Scott-Andrews, Annalise Lane, Sarah Rock, Leah E Robinson

**Affiliations:** 1 School of Kinesiology University of Michigan Ann Arbor, MI United States

**Keywords:** physical activity, motor skills, programs and interventions, social media

## Abstract

**Background:**

Social media may be a powerful platform to combat parents’ and children’s low physical activity levels.

**Objective:**

This study surveyed parents’ beliefs about physical activity in order to expand the extant literature concerning the interest in and the design of an effective and feasible social media physical activity (SMPA) program.

**Methods:**

Primary caregivers (n=250; 215 [86%] mothers, 164 [65.6%] White) of children aged 6-12 years completed an online questionnaire. Interest was examined through responses on the questionnaire; beliefs (ie, perceptions, knowledge, and support) about physical activity were examined using Spearman correlations; and to support the SMPA program design, researchers examined a combination of multiple-choice and free-response questions. For the free-response questions, the researchers performed open coding related to perceived benefits, barriers, and motivators.

**Results:**

Parent respondents (n=215, 86%) were interested in a SMPA program tailored for families. Regarding beliefs, parents exhibited a monotonic relationship between 2 questions related to perceptions of physical activity levels in their children (r_s(250)_=.310, *P*<.001), knowledge about physical activity and motor skills (r_s(250)_=.328, *P*<.001), and support of physical activity and motor skills (r_s(250)_=.385, *P*<.001). Parents perceived benefits of a SMPA program, highlighting family time and health. Barriers included time constraints, a lack of motivation, and environmental factors.

**Conclusions:**

Parents are interested in supporting healthy family behaviors using a SMPA program. An effective program should emphasize motor skill activities, be fun and family oriented, and incorporate incentives, goal setting, and advice and tips. SMPA also needs to address identified barriers, such as those regarding time and environment.

## Introduction

Physical activity is defined as any bodily movement produced by skeletal muscles that results in energy expenditure above the resting metabolic rate [1], often categorized into light (minimal energy expenditure), moderate (requires some effort), and vigorous (activities that lead to harder breathing, puffing, and panting). Currently, physical activity levels are extremely low for children [2] and adults [3] in the United States. Only 42% of children aged 6-11 years achieve the recommended goal of 60 minutes per day of moderate-to-vigorous physical activity (MVPA) each day [2]. In comparison, merely 11% of adults achieve the recommended goal of 150 minutes of MVPA or 75 minutes of vigorous physical activity (VPA) per week [3]. Even further reductions in physical activity levels have been observed in both adults [4] and children [5] during the COVID-19 pandemic, which has limited physical activity opportunities (eg, sports and physical education). Such data are concerning in the light of evidence supporting that low physical activity levels adversely affect physical and mental health [6]. Thus, increasing the avenues for physical activity is a pertinent concern in our evolving society. This need is also supported by Chen et al [7], who highlighted the need for physical activity during the COVID-19 pandemic, with access to programming that is simple and can be completed in the home.

It is imperative to incorporate families in the design and implementation of health programs. Most aspects of health (eg, health socialization, disease prevention, and recuperative care) are centered and accomplished within families [8]. Additionally, children’s lives are generally structured within a family, and children tend to emulate their parents’ (or caregivers’) habits, including physical activity behaviors [9]. Research indicates that physical activity programs that incorporate families, especially those tailored to the participating children, effectively increase physical activity in children [10,11] and adults [12]. Although families are an ideal setting for understanding and intervening on child physical activity, they are complex and multidimensional social groups in terms of their composition, structure, and functions. Defining “family” is challenging because families are diverse, and the term “family” holds different meanings and functions for every individual. In research that focuses on family-based interventions, family is often defined differently but incorporates at least 1 parent and 1 child [12]. It is important to note that as children transition into adulthood, the influence of parents on children’s physical activity decreases [13]. Therefore, family physical activity interventions are generally conducted in children versus adolescents [9,10]. Moreover, family involvement in children’s physical activity programs is a critical determinant and best practice [14,15]. However, despite positive findings, the components of an effective physical activity program for families are not yet agreed upon due to heterogeneity among studies, such as the length of intervention and methodological quality [12].

The use of technology to promote physical activity has become increasingly popular. For example, virtual reality applications, such as Pokémon Go, have been shown to increase physical activity levels of its users by an average of nearly 1500 steps per day in 30 days [16]. More specifically, social media has grown as a flexible, popular platform consisting of social networks, supports, connections, or social interactions [17] among people [18]. The idea that social media is characterized by “user generated” content [17], a term that has existed for at least a decade [17], remains well accepted today [18]. Through social media, users can share information with others, provide social support, and access programs [19]. Although an increasing variety of applications can be considered social media, or used to access social media, only applications and platforms with the qualities mentioned earlier are considered social media for this paper’s purposes. It should be noted that this excludes virtual reality and exergaming applications [20]. Under this definition, some of the most popular platforms are Facebook, YouTube, and Instagram [21]. In the past 15 years, adult Americans' social media usage increased from 5% usage in 2005 to approximately 72% in February 2019 [21]. Researchers have begun leveraging these cost-effective cyber environments to promote health behaviors and aid in behavior change [19]. However, social media has not been highly utilized to target low levels of physical activity [19,22,23]. More insight related to social media usage and perceptions of target audiences is needed to inform the development of a program delivered to families via social media platforms that are designed to increase physical activity in both parents and their children (ie, a social media physical activity [SMPA] program for families).

From a feasibility standpoint, it is also critical to measure parental interest in a SMPA program, understand what families believe will support their engagement in such a program, and incorporate these factors into program design. However, little research has used and assessed SMPA programs [22,23], with most queries focused on internet- and technology-based programs [12,24]. Robbins et al [22] evaluated the feasibility, acceptability, and preliminary efficacy of a 12-week social media intervention on adolescents’ (aged 10-13 years) BMI, physical activity, and diet quality [22]. The intervention included an after-school club for adolescents, parent-adolescent meetings, and a parent Facebook group. This study found that adolescents had significantly greater autonomous motivation for physical activity after completing the intervention. Additionally, Wojcicki et al [23] investigated the feasibility of an 8-week Facebook physical activity intervention for teenagers (aged 13-15 years) exhibiting low levels of physical activity. The intervention consisted of access to a private Facebook group with 2 daily wall posts containing youth-based physical activity information and resources [23]. There were no changes in objective physical activity (ie, measured by an accelerometer); however, adolescents reported increases in subjective physical activity (ie, self-reported) [23]. In both these studies, Facebook was a feasible delivery mode and was supported by both parents and children.

Physical activity programs for families have been delivered online or through eHealth applications. However, it is essential to note that these programs do not incorporate social media elements, such as user-generated information, social support, or participant interaction [12,24]. In a systematic review of physical activity programs for families, 6 (13%) of the 47 studies were delivered online and 1 (2%) communicated with participants through email [12]. Notably, 4 (67%) of the 6 studies cited positive effects and 5 (83%) of the 6 studies were favored by participants [12]. Another systematic review specifically examined eHealth programs for families, and 6 (86%) of the 7 studies included had additional components besides online delivery (eg, face-to-face and telephonic components) [24]. Interestingly, the 1 (14%) study that was conducted entirely online was the only one that found significant BMI changes [24]. Even though only 1 (14%) study found significant effects, overall study participants favored the internet as a medium for health programs [24]. These findings demonstrate that online programs tailored for families are feasible and highly favored, but social media has not been a widely utilized platform.

Given that SMPA programs have not been broadly implemented for families, it is vital to understand parental interest and program design considerations to support buy-in for a SMPA program. In addition to understanding parents’ interest in a SMPA program, it is also critical to understand their viewpoints on such programs’ targeted behaviors that are critical to children’s healthy growth and development, specifically physical activity and secondarily gross motor skills. Motor skills are defined as the “building blocks” of more advanced, complex movements required to participate in sports, games, or other physical activities [25]. Motor skills are included as a secondary targeted behavior of a physical activity program because of their essential role in supporting movement and their associations with positive trajectories of health [26]. Motor skills are defined as movement behaviors required to participate in sports, games, and other context-specific physical activities [26]. The literature supports that proficiency in a wide range of motor skills, often called motor competence, is needed to support lifelong movement and physical activity [26]. Motor skills are positively associated with physical activity, health-related fitness, perceived motor competence, weight status, and academic performance in children [26].

The research on parents’ beliefs of their children’s physical activity and motor skills is sparse [27], and the limited findings in this area are mixed [28-35]. Evidence supports that those parents who are knowledgeable about physical activity and motor skills are more likely to support these behaviors in their children [28,29] and that their children are more likely to have greater motor abilities [30]. However, research has found that parents hold inaccurate perceptions about physical activity and motor skills, as they tend to overestimate their children’s physical activity [31-33] and motor abilities [34,35]. More research is needed to measure parents’ beliefs of their child’s engagement in motor skills and physical activity in order to determine the extent to which families believe that participating in a physical activity program is essential and would be beneficial.

This study aims to (1) gauge interest in a SMPA program delivered to families, (2) examine parental beliefs about physical activity (as a proxy measure for buy-in to a SMPA program), and (3) gather information to inform the design of SMPA programs to best suit the needs of their participants, thereby maximizing efficacy and feasibility. These aims were addressed by a questionnaire designed to elucidate the relationship between parental beliefs about physical activity and motor skills in their children and gain insight into the benefits parents perceived could be gleaned from program participation and the motivators/barriers parents perceived to program engagement.

## Methods

### Study Approval

The institutional review board (health sciences and behavioral sciences) at the University of Michigan reviewed this study and approved it with exemption (HUM00161089). The study was conducted entirely online, and no identifying data were collected.

### Measures

Experts in the fields of kinesiology and public health developed an online questionnaire to assess parent beliefs. The group of 3 experts included a professor of kinesiology with over 15 years of experience working in the field of motor development who conducts motor skill interventions in children, a professor of health behavior research who has more than 10 years of experience in scientific and clinical aspects of behavioral medicine and public health, and a PhD candidate who holds a Certified Health Education Specialist (CHES) certification and a master’s degree in health behavior and health education with over 6 years of experience working with children. The questionnaire was developed through extensive literature review and screening, piloting to families, and reworking. The questionnaire consisted of 42 questions (ie, 39 multiple-choice and 3 free-response questions) divided into 3 sections: demographics, beliefs about physical activity and motor skills, and social media use and interest in a SMPA program. The questionnaire was distributed through an online platform Qualtrics (Provo, UT, USA) and took approximately 23 minutes to complete. It should be noted that the survey inquired about parents’ interest in a SMPA program for families, but there was no clear definition for what this social media program would look like nor was a definition of “family” provided. We sought to receive input about program design from their perspective and allow parents to offer opinions and ideas unrestrictedly.

### Participants

Participants were a convenience sample who self-selected to participate in this study. Participants were recruited in the summer of 2019 through flyers placed around the community, a posting on the university research registry, and emails sent to a listserv for a local summer camp program. The questionnaire was available from June to August 2019. Inclusion criteria were being the parent or primary guardian of a child aged 6-12 years and residing in Michigan.

### Data Analyses

Quantitative data were analyzed using IBM SPSS Statistics (version 26), with α levels set to .05 a priori. Qualitative data were analyzed using qualitative analysis software NVivo 12 (QSR International, Doncaster, Australia). Data analysis was conducted using the grounded theory methodology outlined by Charmaz [36], utilizing line-by-line coding and constant comparative methods. This process involves taking an inductive approach to understanding and learning from the data. Coding was conducted by the first and third authors (KS and SR). The authors first engaged in line-by-line coding of all the responses, developed focused codes, and then derived themes about each of the 3 questions’ responses, comparing across all responses. The 2 authors had 91% agreement in coding across the 3 qualitative questions analyzed (ie, question about benefits, 92%; question about barriers, 91%; and question about motivation, 90%). Discrepancies were discussed and resolved among the 2 authors.

#### Interest

Interest was examined using percentages of responses to the 2 multiple-choice questions, *Are you interested in participating in a family-based physical activity program through social media?*, with the choices being *not interested*, *somewhat interested*, and *very interested*, and *What do you usually do on the internet? Check all that apply. (Email, browse on the web, social media, text messaging, other)*.

#### Beliefs About Physical Activity and Motor Skills

Spearman correlations were used to examine beliefs about physical activity and motor skills or specifically the associations between multiple-choice questions asking about parents’ perceptions, knowledge, and support of physical activity and motor skills: values of ≥.80 were considered very strong; .60-.79, strong; .40-.59, moderate; .20-.39, weak; and 0-.19, very weak [37]. For parental perceptions, the association between the 2 questions *How physically active would you say your child is?*, measured on a 5-point Likert scale (eg, very inactive to very active), and *Do you think your child needs to be more physically active?*, measured with a dichotomous response (ie, yes or no), was examined. To compare these questions, responses to the former question was dichotomized into 2 variables (ie, active and inactive), with the neutral statement being categorized as inactive. Follow-up sensitivity analyses were conducted to examine the effect on Spearman correlation results if neutral answers were categorized as active instead of inactive. For parental knowledge, the association between the 2 questions *Do you think your child needs to be more physically active?* and *Do you think your child needs improvements in their motor skills?*, both measured dichotomously, was examined. Parental support of physical activity and motor skills was analyzed via the association between the 2 questions *How often do you give your child opportunities to engage in physical activity?* and *How often do you encourage your child to develop motor skills?* Possible answer choices to both questions were *every day*, *2-3 times per week*, *once a week*, *a few times a month*, *once a month*, and *never*.

#### Program Design

Program design was examined through 3 multiple-choice questions and coding of 3 free-response questions to help inform the program design for SMPA programs. The 3 multiple-choice questions were *How often did you use social media platforms (Facebook, Twitter, Instagram, texting apps, and fitness apps) in the past month?*, with choices being *never*, *rarely*, *sometimes*, *very often*, and *always*; *What information or content would be most helpful for your family?*, with choices being *goal setting*, *family activities*, *advice and tips*, *educational videos*, *social support*, and *other*; and *How would you like to receive this information?*, with choices being *email*, *text messages*, *social media messaging*, *social media group*, and *other*. Participants were permitted to select multiple choices for the latter 2 questions. The 3 coded free-response questions were *What are some of the benefits you foresee for your family to be part of a social media–based physical activity and fundamental motor skill program?*, *What are some of the barriers you foresee for your family to be part of a social media–based physical activity and fundamental motor skill program?*, and *What would motivate you and your family to be involved in a social media–based physical activity and fundamental motor skill program?*.

## Results

### Participant Details

A total of 335 participants started the questionnaire. Of these, 65 (19.4%) were removed because they completed less than 34% of the questionnaire (ie, completed only the demographic section or less), 9 (2.7%) whose child was not within the age range were removed, and 11 (3.3%) who did not reside in Michigan were removed. There were a total of 250 primary caregivers (215 [86%] mothers, 105 [42%] aged 30-39 years; see Table 1) included in the data analyses. Children of parent respondents had a mean age of 8.7 years, and 139 (55%) were girls.

**Table 1:** Sociodemographic characteristics of participants (N=250).

Characteristics		n (%)
**Relationship to the child**		
	Mother	215 (86.0)
	Father	18 (7.2)
	Grandparent	3 (1.2)
	Legal guardian	6 (2.4)
	Other	8 (3.3)
**Parents’ age (years)**		
	20-29	30 (12.0)
	30-39	105 (42.0)
	40-49	90 (36.0)
	50-59	21 (8.4)
	60-69	3 (1.2)
	≥70	1 (0.4)
**Child’s race/ethnicity**		
	White	164 (65.6)
	Hispanic or Latinx	13 (5.2)
	Black or African American	26 (10.4)
	Native American Indian	1 (0.4)
	Asian	5 (2.4)
	Other/biracial	40 (16)
**Parents’ highest level of education**		
	Less than high school degree	5 (2.0)
	High school degree or equivalent	14 (5.6)
	Some college but no degree	46 (18.4)
	Associate degree	25 (10.0)
	Bachelor’s degree	69 (27.6)
	Graduate degree	87 (34.8)
	Other	4 (1.6)
**Total number of adults in the household**		
	1	30 (12.1)
	2	190 (76.3)
	3	24 (9.6)
	4	4 (1.6)
	≥5	1 (0.4)
**Total household income (US $)**		
	≤24,999	34 (13.6)
	25,000-49,999	42 (16.8)
	50,000-99,999	74 (29.6)
	100,000-149,999	49 (19.6)
	≥150,000	50 (20.0)
**Total number of children in the household**		
	1	48 (19.2)
	2	104 (41.6)
	3	51 (20.4)
	4	26 (10.4)
	≥5	21 (8.4)

#### Interest

Of the 250 parents who completed the survey, 214 (85.6%) were interested in a family-based SMPA program. Of these, 97 (45.2%) were somewhat interested and 86 (40.4%) were very interested, while 178 (83.2%) of parents reported that they usually engage in social media use.

#### Beliefs About Physical Activity and Motor Skills

There was a weak but significant positive association between the 2 questions on parents’ perceptions of their child’s physical activity (r_s(250)_=.310, *P*<.001). We found that 204 (81.6%) of the 250 parents responded that their child was active and 125 (50%) responded that their child needs to improve their physical activity levels. When neutral answers were categorized as active instead of inactive for the sensitivity analysis, there was a slight change in the Spearman correlation (r_s(250)_=.136, *P*<.03). Although dichotomizing the neutral answers in the opposite direction did produce slightly different statistics, the primary findings remained the same. There was a weak but significant positive association between the questions regarding parents’ knowledge of their child’s physical activity and motor skills (r_s(250)_=.328, *P*<.001). We found that 125 (50%) of the parents responded that their child does not need to be more physically active, and 131 (52.4%) of the parents responded that their child does not need to improve motor skills. Additionally, 220 (88%) of the parents acknowledged a difference between physical activity and motor competence, and 249 (99.6%) responded that motor competence supports healthy development. There was also a weak but significant positive association between the questions about parents’ support of their child’s physical activity and motor skill behaviors (r_s(250)_=.385, *P*<.001). We found that 207 (82.8%) of the parents reported providing their child with physical activity opportunities every day, while only 136 (54.5%) of the parents reported providing their children with motor skill opportunities every day. In addition, 21 (8.4%) of the parents responded that they encourage motor skills a few times a month to never, while only 2 (0.8%) of the parents responded that they promote physical activity a few times a month to never.

#### Program Design

#### Quantitative Feedback

In response to the questions about social media use, 212 (84.8%) of the 250 parents responded that within the past month, they use Facebook *sometimes*, *very often*, or *always*. Within the past month, 209 (83.6%) of the parents responded that they used texting apps (eg, text messaging, iMessage, WeChat, and WhatsApp) *sometimes*, *very often*, or *always*. Parents also indicated that they used fitness apps, such as MyFitnessPal, Strava, RunKeeper, and Nike Training Club (n=112, 44.8%); Instagram (n=96, 38.4%); and Twitter (n=41, 16.4%) *sometimes*, *very often*, or *always*. In response to the questions regarding SMPA program content, parents responded that it would be helpful to provide materials and information regarding *goal setting* (n=154, 61.6%), *family activities* (n=191, 76.4%), *advice and tips* (n=133, 53.2%), *social support* (n=90, 36%), and *educational videos* (n=86, 34.4%)*.* Regarding how often parents would like to receive physical activity and motor skills content, 137 (54.9%) of the parents preferred *email*, 85 (34%) preferred *social media groups*, 82 (32.8%) preferred *text messages*, and 45 (18%) preferred *social media messaging*.

#### Qualitative Feedback

Qualitative analysis of the 3 free-response questions yielded predominant themes about program design, including benefits, barriers, and motivators (see Table 2). Parents who responded to the question *Do you think your child needs to be more physically active?* with yes or no both contributed equally (n=123 [49%] said yes) to the qualitative responses. For question 9, regarding benefits, 7 themes were derived from 212 responses: *family time*, *health improvement*, *accountability and motivation*, *fun and enjoyment*, *community relationships*, *modeling*, and *no benefit*. Within these responses, 128 (60.4%) were related to the theme of *family time*, 112 (52.8%) to *health improvement*, 37 (17.5%) to *accountability*, 28 (13.2%) to *fun and enjoyment*, 18 (8.5%) to community relationships, 17 (8%) to modeling, and 7 (3.3%) to *no benefit*. Question 10, concerning barriers, revealed 7 main themes from a total of 207 responses: *time*, *environment*, *motivation and interest*, *technology*, *health concern*, *money issues*, and *no barrier*. Within these responses, 137 (66.2%) encompassed the theme of *time*, 60 (28.9%) encompassed *motivation and interest*, 56 (27.1%) encompassed *environment*, 19 (9.2%) encompassed *technology*, 8 (3.9%) encompassed *health concern*, 7 (3.4%) encompassed *money issues*, and 12 (5.8%) encompassed *no barrier*. From Question 11, about motivators, 8 prominent themes arose from 195 total responses: *social support*, *health benefits*, *incentive*, *tracking and goal setting*, *cost*, *ease of use and access*, *fun and competition*, and *does not know*. Among these responses, 70 (35.9%) were related to *incentives*, 52 (26.7%) to *fun and competition*, 39 (20%) to *social support*, 26 (13.3%) to ease of use and access, 24 (12.3%) to *health benefits*, 20 (10.3%) to *tracking and goal setting*, 18 (9.2%) to *does not know*, and 7 (3.6%) to *cost*.

**Table 2:** Interest in a social media program.

Theme			Definition		Examples		Responses, n (%)
**Question 9. What are some of the benefits you foresee for your family to be part of a social media–based physical activity and fundamental motor skill program? (N=212)**							
	Family time	Building better family relationships and spending more quality time together		*Spending time together would be number one. We love to connect as a family.*		128 (60.4)	
	Health improvement	An array of health benefits, such as increasing physical activity, overall health, endurance, and strength		*Getting healthy together.* *Better mental and physical health.*		112 (52.8)	
	Accountability and motivation	A way to be held to a certain standard or be motivated to participate in certain tasks		*Having something for the whole family keeps everyone accountable.*		37 (17.5)	
	Fun and enjoyment	A fun way to spend time with family members and bring happiness to those involved		*It seems like it would be more fun to do it together. We could motivate and encourage each other.*		28 (13.2)	
	Community relationships	Creating new relationships and friends with community members		*Connecting with other families, a sense of community.* *Creating new friendships.*		18 (8.5)	
	Modeling	Demonstrating the importance of physical activity		*Showing that being active is important for adults and kids.* *Being a role model.*		17 (8.0)	
	No benefit	Not foreseeing any benefit to the program		*I don’t think we could benefit from social media activities.*		7 (3.3)	
**Question 10. What are some of the barriers you foresee for your family to be part of a social media–based physical activity and fundamental motor skill program? (N=207)**							
	Time	An inability to find time to participate in the program due to school/work schedules		*Finding the time and weather are our biggest barriers.*		137 (66.2)	
	Motivation and interest	A lack of motivation, interest, or energy due to busy lives		*My child not being interested, me losing motivation.*		60 (28.9)	
	Environment	Environmental factors that are a concern, such as location, weather, and access to safe areas		*We do not have sidewalks where we live.*		56 (27.1)	
	Technology	Issues with technology, privacy, or social media		*Invasion of privacy.* *Our kids are not on social media yet, due to age.*		19 (9.2)	
	Health concern	Underlying health conditions that could be a problem when participating		*My current fitness/health level not being optimal.*		8 (3.9)	
	Money issues	The price of the program as a financial barrier		*Extra costs will affect our ability to participate.*		7 (3.4)	
	No barrier	No barriers foreseen		*We are active already.*		12 (5.8)	
**Question 11. What would motivate you and your family to be involved in a social media based–physical activity and fundamental motor skill program? (N=195)**							
	Incentive	A tangible object/monetary reward as compensation for completing portions of the program		*Some sort of reward would be the highest motivator.* *Maybe earning rewards or “badges.”*		70 (35.9)	
	Fun and engaging	Incorporating aspects of fun and competition into the program to make it more enticing to participants		*If my child sees it as fun.* *It has to be engaging.*		52 (26.7)	
	Social support	Increased support from a social circle that allows participants to connect in new ways		*I think if there were accountability partners, I would be motivated to participate. Friends have a way of keeping you honest.*		39 (20.0)	
	Ease of use and access	Allowing the program to be easy to use, accessible to all, and carefully planned out		*Knowing more about the program and being aware of what was going to be posted ahead of time.*		26 (13.3)	
	Health benefits	Health improvements from participation		*Seeing how it could keep my child healthy.* *Improving our health.*		24 (12.3)	
	Tracking and goal setting	Using various tracking and goal-setting mechanisms to actively see progress over time		*Goal setting with daily/weekly check ins.* *Keeping track of progress.*		20 (10.3)	
	Does not know	Uncertain or unsure of what would motivate the participants		*Not sure.*		18 (9.2)	
	Cost	Making the price of the program fair and flexible for participants		*The program is free and interesting to kids and adults.*		7 (3.6)	

## Discussion

### Principal Findings

The purpose of this study was to gauge interest in a SMPA program for families, examine parental beliefs about physical activity and motor skills, and gather information to inform the design of a SMPA program. Through the use of an online questionnaire, it was found that a large majority of parents (214/250, 85.6%) are interested in a family-based SMPA program. Previous research supports our findings—that parents and children favor online physical activity programs, including SMPA programs for families [12,22-24].

This study found that parents hold accurate beliefs based on their perceptions, knowledge, and support of physical activity and motor skills. Parents had accurate perceptions that their child’s physical activity and motor skills are associated, demonstrated knowledge that there is a difference between motor skills and physical activity, and supported physical activity and motor skills. Despite a large number of responses indicating that parents thought their child achieves the daily amount of physical activity recommended, it is important to note that approximately 125 (50%) of the parents believed that their child needed to increase their physical activity levels or motor skills. Responses indicated that at least half (n=125, 50%) of the parents had the motive to buy-in to a SMPA program. Based on research illustrating low levels of physical activity [2] and motor skills [38] in children, and that parents overestimate their children’s physical activity levels [31-33] and motor skills [34,35], it is likely that some parents perceive their children as more active than they actually are or to have more advanced motor skills than they actually do. Thus, it can be reasonably assumed that the percentage of parents whose children need to increase their physical activity levels or motor skills to meet national recommendations [2] is more significant than this study found.

Based on the findings that more parents provide their children with daily physical activity opportunities compared to motor skill opportunities (n=207 [82.8%] vs n=136 [54.5%]), and that a larger percentage of parents indicated that their child needed to be more physically active relative to the percentage of parents who indicated that their child needed to improve their motor skills (n=125 [50%] vs n=119 [47.6%]), it appears that parents may regard physical activity as more important than motor skills. Interestingly, 220 (88%) of the 250 parents acknowledged a difference between physical activity and motor competence, and 249 (99.6%) correctly responded that motor skills support healthy development. These findings are supported by a recent study that examined the relationship between motor skills and the home environment, which found that parents recognize motor skills as critical underlying factors regarding physical activity [30]. These findings suggest that parents may intend to provide their children with the same quantity of motor skill development opportunities as physical activity opportunities. Still, they lack the knowledge regarding how to do so. Although research is limited on parents’ knowledge of physical activity and motor skills [27], we know that knowledgeable parents are more likely to support physical activity and motor skills in their children [28,29] and parents of children who value motor skills have higher motor skill proficiency [30]. Such research emphasizes the importance of educating parents about motor skills and providing motor skill opportunities for their children.

Parents’ responses to the various closed and open-ended questions provide key elements that should be incorporated into the program design of a SMPA program. First, given the importance of motor skills [26] and the finding that physical activity opportunities are more commonly provided than motor skill opportunities, a SMPA program must include components that will educate parents about motor skills and incorporate motor skill opportunities. Next, most parents responded that content focusing on family activities would be helpful and that family time would benefit from a SMPA program. About half of the parents (133/250, 53%) responded that they are interested in *health* components as well. However, previous research supports that physical activity programs may be efficacious to emphasize components and content unrelated to weight loss or health improvement [12]. Thus, SMPA should predominately feature fun and family-oriented content. Lastly, parents indicated that they would like the content of a SMPA program to focus on goal setting and program advice and tips. Incorporating goal setting and program advice and recommendations has been shown to be successful in previous online interventions for children [24]. Thus, we recommend that a SMPA program include fun activities that promote the use of motor skills, involve multiple or all family members to encourage together-time, and deliver goal-setting prompts and tips to help families maximize participation in and benefit from the program.

Parents also highlighted barriers and motivators that should be incorporated into program design. The most prevalent barriers to program participation that parents identified were time, motivation and interest, and environmental factors. Parents noted being unable to find the time to complete the program, having a lack of motivation, and limitations due to environmental factors, such as cold winter weather and a lack of safe outdoor space. Thus, a SMPA program should feature physical activity and motor skill activities of various durations and include workouts that can be done both indoors and in a variety of outdoor environments, especially given that access to exercise facilities/opportunities has been shown to dictate the use of these environments for exercise and physical activity [39]. Environmental factors impacting Michiganders’ participation in a SMPA program may differ from families in other regions. A program that is fun and engaging and promotes together-time, as suggested earlier, as well as one that considers various environmental and time constraints, is likely to consequently minimize the barrier of motivation and interest. With regard to motivators to program participation, incentives, fun and engaging, and social support were among the top 3 themes of free-response answers. Incorporating rewards into physical activity interventions for families has previously been found to significantly increase the pedometer step count in children compared to the control group [40]. It has been suggested as a means of augmenting motivation elsewhere [12].

Further, our study found that parents participating in the questionnaire were already active on varying social media platforms, with Facebook being the most often utilized. Our results align with Pew Research that social media is popular among adults and that Facebook is the most utilized platform [21]. However, Pew Research cited that Instagram and YouTube were the subsequent 2 most utilized platforms [21]. In this study, only 96 (38.5%) of the 250 parents reported using Instagram *sometimes*, *often*, and *always.* Although a specific option to choose YouTube was not included, participants were provided an opportunity to name any social media platform for the question *How would you like to receive this information?* [21]. However, no participants wrote YouTube. The finding that parents were highly active on social media supports the idea that Facebook is a promising and favorable mechanism to deliver a SMPA program, which has been previously established [19,22,23]. However, participants of this study demonstrated a preference for email delivery of physical activity and motor skill programs when asked directly. This platform is easily accessible for parents, and children do not necessarily have to be on social media to participate in the program, as the mode of delivery would be via parents. Given that a wide variety of social media platforms have been found to be effective in eHealth interventions, such as text messaging, web-based chat groups, and mobile phone applications [24], any form of social media used in a SMPA program would likely be both accessible and successful in the dissemination of physical activity and motor skill program content. Finding the best social media platform for such a program should involve both parents and children, as they are the stakeholders [41].

Importantly, this study shows that there is both widespread interest in and potential program buy-in for the largely unstudied concept of a SMPA program for families, particularly among a Michigan sample of parents. We also interpreted various novel feedback and insight about a SMPA program specifically tailored to families into meaningful advice for researchers seeking to design such a program. In particular, we found that a SMPA program should include motor skill–focused educational content, activities that promote together-time, advice and tips, and prompts for regular goal setting, environmentally and time conscious workouts, and incentives. Ideal mediums for content delivery were found to be Facebook and email.

### Strengths

The strengths of this study include a large, diverse sample of 250 participants whose characteristics of ethnicity and income brackets aligned with US Census data [42] for Michigan. It is important to note that our sample does not align as well with national US Census data and the National Health and Nutrition and Examination Survey, as we had overrepresentation of Whites and a lower representation of Hispanic or Latinx and Asians [43,44]. Another strength is that independent coders were used to examine the free-response questions. This study aimed to understand the feasibility of a SMPA program designed based on families’ needs.

### Limitations

This study was conducted using an online questionnaire through Qualtrics. There are advantages and disadvantages to using an online platform for data collection. A large, diverse sample of participants from across Michigan was able to be obtained. Since the data were collected via a convenience sample, it is important to acknowledge that it is possible that the questionnaire was completed by parents who have a high level of knowledge about and support of motor skills and physical activity regarding healthy development or whose children engage in above-average levels of physical activity or motor skills. We understand that the recruiting methods may have biased the type of parents who responded [39]. It is commonly known that participants of higher socioeconomic status and higher education levels may have more knowledge and opportunities.

Further, we acknowledge that gauging interest in a program that offers appealing health and activity benefits is likely to yield high interest rates, as seen in this study. However, given that interest rates are also likely influenced by perceptions of exercise, which can often be negative [45], the authors feel the interest rates presented are not biased. Additionally, since all data were collected via a questionnaire, no comparisons to actual physical activity levels and motor skill abilities could be made. Caution must be taken in generalizing the results of this study, given that the sample was limited to Michigan residents and not fully representative of national demographics of parents of children aged 6-12 years. It is recommended that future research be conducted on a broader scale to expand the understanding of feasibility and interest in SMPA programs for families in populations this study did not adequately represent. A larger-scale study is critical important, given that the perceptions of and the ability to participate in physical activity can vary depending upon social determinants of health, as well as race and ethnicity [46,47]. Nevertheless, these findings will meaningfully assist in the development of a SMPA program.

### Conclusion

Social media has become a popular medium for communication and information dissemination over the past 15 years [21]. The current COVID-19 pandemic has particularly emphasized the importance of technology, including social media, to facilitate social connections and engagement in different health behaviors. The benefits of social media may be a powerful tool to support a physical activity program for families [19]. This study found a need for the development of a social media program to support families’ physical activity. This study also found that an effective SMPA program should emphasize motor skill activities, be family oriented, and incorporate incentives, goal setting, and advice and tips. A SMPA program must be developed with identified barriers, such as the environment (eg, weather, space, and accessibility), time (eg, duration and ease of use), and type of program (eg, fun and engaging), in mind. Future research and program development should continue to centralize best practices and rigor while tailoring programs to the needs of those receiving them [9].

## Abbreviations

MVPA: moderate-to-vigorous physical activity
SMPA: social media physical activity
VPA: vigorous physical activity

## References

[1] Caspersen CJ, Powell KE, Christenson GM (1985). Physical activity, exercise, and physical fitness: definitions and distinctions for health-related research. Public Health Rep.

[2] Troiano R, Berrigan D, Dodd K, Mâsse LC, Tilert T, McDowell M (2008). Physical activity in the United States measured by accelerometer. Med Sci Sports Exerc.

[3] Tucker J, Welk G, Beyler N (2011). Physical activity in U.S.: adults compliance with the Physical Activity Guidelines for Americans. Am J Prev Med.

[4] Puccinelli P, da Costa TS, Seffrin A, de Lira CAB, Vancini RL, Nikolaidis PT, Knechtle B, Rosemann T, Hill L, Andrade MS (2021). Reduced level of physical activity during COVID-19 pandemic is associated with depression and anxiety levels: an internet-based survey. BMC Public Health.

[5] Dunton G, Do B, Wang S (2020). Early effects of the COVID-19 pandemic on physical activity and sedentary behavior in children living in the U.S. BMC Public Health.

[6] US Department of Health and Human Services (2018). Physical Activity Guidelines for Americans. 2nd ed.

[7] Chen P, Mao L, Nassis G, Harmer P, Ainsworth B, Li F (2020). Coronavirus disease (COVID-19): the need to maintain regular physical activity while taking precautions. J Sport Health Sci.

[8] Hanson C, Crandall A, Barnes M, Magnusson B, Novilla M, King J (2019). Family-focused public health: supporting homes and families in policy and practice. Front Public Health.

[9] Hutchens A, Lee (2018). Parenting practices and children's physical activity: an integrative review. J Sch Nurs.

[10] Rhodes R, Blanchard C, Quinlan A, Naylor P, Warburton D (2019). Family physical activity planning and child physical activity outcomes: a randomized trial. Am J Prev Med.

[11] Chen J, Weiss S, Heyman M, Cooper B, Lustig RH (2011). The efficacy of the web-based childhood obesity prevention program in Chinese American adolescents (Web ABC study). J Adolesc Health.

[12] Brown H, Atkin A, Panter J, Wong G, Chinapaw M, van Sluijs EMF (2016). Family-based interventions to increase physical activity in children: a systematic review, meta-analysis and realist synthesis. Obes Rev.

[13] Pate R, Dowda M, Dishman R, Colabianchi N, Saunders R, McIver KL (2019). Change in children's physical activity: predictors in the transition from elementary to middle school. Am J Prev Med.

[14] Pratt K, Cotto J, Goodway J (2017). Engaging the family to promote child physical activity. ACSMs Health Fit J.

[15] Horodyska K, Luszczynska A, van den Berg M, Hendriksen M, Roos G, De Bourdeaudhuij I, Brug J (2015). Good practice characteristics of diet and physical activity interventions and policies: an umbrella review. BMC Public Health.

[16] Althoff T, White RW, Horvitz E (2016). Influence of Pokémon Go on physical activity: study and implications. J Med Internet Res.

[17] Kaplan A, Haenlein M (2010). Users of the world, unite! The challenges and opportunities of social media. Bus Horiz.

[18] Verma S, Yadav N (2021). Past, present, and future of electronic word of mouth (EWOM). J Interact Mark.

[19] Moorhead S, Hazlett D, Harrison L, Carroll J, Irwin A, Hoving C (2013). A new dimension of health care: systematic review of the uses, benefits, and limitations of social media for health communication. J Med Internet Res.

[20] Siani A, Marley SA (2021). Impact of the recreational use of virtual reality on physical and mental wellbeing during the Covid-19 lockdown. Health Technol (Berl).

[21] Demographics of Social Media Users and Adoption in the United States.

[22] Robbins LB, Ling J, Clevenger K, Voskuil VR, Wasilevich E, Kerver JM, Kaciroti N, Pfeiffer KA (2020). A school- and home-based intervention to improve adolescents' physical activity and healthy eating: a pilot study. J Sch Nurs.

[23] Wójcicki TR, Grigsby-Toussaint D, Hillman CH, Huhman M, McAuley E (2014). Promoting physical activity in low-active adolescents via Facebook: a pilot randomized controlled trial to test feasibility. JMIR Res Protoc.

[24] Hammersley M, Jones R, Okely A (2016). Parent-focused childhood and adolescent overweight and obesity eHealth interventions: a systematic review and meta-analysis. J Med Internet Res.

[25] Gallahue D, Ozmun JC, Goodway J (2012). Understanding Motor Development: Infants, Children Adolescents, Adults.

[26] Robinson L, Stodden D, Barnett L, Lopes VP, Logan SW, Rodrigues LP, D'Hondt E (2015). Motor competence and its effect on positive developmental trajectories of health. Sports Med.

[27] Thompson J, Jago R, Brockman R, Cartwright K, Page A, Fox K (2010). Physically active families: de-bunking the myth? A qualitative study of family participation in physical activity. Child Care Health Dev.

[28] Haddad J, Ullah S, Bell L, Leslie E, Magarey A (2018). The influence of home and school environments on children's diet and physical activity, and body mass index: a structural equation modelling approach. Matern Child Health J.

[29] Sawyer A, Smith L, Schrempft S, van Jaarsveld CHM, Wardle J, Fisher A (2014). Primary caregiver knowledge of paediatric physical activity recommendations in the United Kingdom and its association with caregiver behaviour: an observational study. BMC Public Health.

[30] Jarvis S, Williams M, Rainer P, Saunders J, Mullen R (2020). The relationship of family characteristics, parental beliefs and parenting behaviours with the fundamental movement proficiency of primary school children in South East Wales. Eur Phys Educ Rev.

[31] Corder K, Crespo N, van Sluijs EMF, Lopez N, Elder J (2012). Parent awareness of young children's physical activity. Prev Med.

[32] Corder K, van Sluijs EM, McMinn AM, Ekelund U, Cassidy A, Griffin SJ (2010). Perception versus reality: awareness of physical activity levels of British children. Am J Prev Med.

[33] Greca J, Arruda G, Coledam D, Pires Junior R, Teixeira M, Oliveira A (2016). Student and parental perception about physical activity in children and adolescents. Rev Andal Med Deporte.

[34] Silva S, Flôres FS, Corrêa SL, Cordovil R, Copetti F (2017). Mother's perception of children's motor development in Southern Brazil. Percept Mot Skills.

[35] Zysset A, Kakebeeke T, Messerli-Bürgy N, Meyer AH, Stülb K, Leeger-Aschmann CS, Schmutz EA, Arhab A, Ferrazzini V, Kriemler S, Munsch S, Puder JJ, Jenni OG (2018). The validity of parental reports on motor skills performance level in preschool children: a comparison with a standardized motor test. Eur J Pediatr.

[36] Charmaz K (2014). Constructing Grounded Theory: 2nd Edition.

[37] Wuensch KL, Evans JD (1996). Straightforward statistics for the behavioral sciences. J Am Stat Assoc.

[38] Brian A, Pennell A, Taunton S, Starrett A, Howard-Shaughnessy C, Goodway JD, Wadsworth D, Rudisill M, Stodden D (2019). Motor competence levels and developmental delay in early childhood: a multicenter cross-sectional study conducted in the USA. Sports Med.

[39] Giles-Corti B, Donovan R (2002). The relative influence of individual, social and physical environment determinants of physical activity. Soc Sci Med.

[40] Finkelstein E, Tan Y, Malhotra R, Lee C, Goh S, Saw S (2013). A cluster randomized controlled trial of an incentive-based outdoor physical activity program. J Pediatr.

[41] Rhodes R, Naylor P, McKay H (2010). Pilot study of a family physical activity planning intervention among parents and their children. J Behav Med.

[42] US Census Bureau QuickFacts: Michigan.

[43] US Census Bureau QuickFacts: United States.

[44] Centers for Disease Control and Prevention National Health and Nutrition Examination Survey: NHANES 2017-2018 Demographics Data.

[45] Nelson T, Benson E, Jensen C (2010). Negative attitudes toward physical activity: measurement and role in predicting physical activity levels among preadolescents. J Pediatr Psychol.

[46] Barr-Anderson D, Flynn J, Dowda M, Taverno Ross SE, Schenkelberg M, Reid L, Pate R (2017). The modifying effects of race/ethnicity and socioeconomic status on the change in physical activity from elementary to middle school. J Adolesc Health.

[47] Ball K, Carver A, Downing K, Jackson M, O'Rourke K (2015). Addressing the social determinants of inequities in physical activity and sedentary behaviours. Health Promot Int.

